# Neuronal ICAM-5 Plays a Neuroprotective Role in Progressive Neurodegeneration

**DOI:** 10.3389/fneur.2019.00205

**Published:** 2019-03-12

**Authors:** Katharina Birkner, Julia Loos, René Gollan, Falk Steffen, Beatrice Wasser, Tobias Ruck, Sven G. Meuth, Frauke Zipp, Stefan Bittner

**Affiliations:** ^1^Department of Neurology, Focus Program Translational Neuroscience (FTN) and Immunotherapy (FZI), Rhine Main Neuroscience Network (rmn2), University Medical Center of the Johannes Gutenberg University Mainz, Mainz, Germany; ^2^Department of Neurology, University of Muenster, Muenster, Germany

**Keywords:** T cells, experimental autoimmune encephalomyelitis, multiple sclerosis, neuroinflammation, adhesion molecules

## Abstract

Multiple sclerosis (MS) is a chronic autoimmune disease of the central nervous system (CNS) leading to CNS inflammation and neurodegeneration. Current anti-inflammatory drugs have only limited efficacy on progressive neurodegenerative processes underlining the need to understand immune-mediated neuronal injury. Cell adhesion molecules play an important role for immune cell migration over the blood-brain barrier whereas their role in mediating potentially harmful contacts between invading immune cells and neurons is incompletely understood. Here, we assess the role of the CNS-specific neuronal adhesion molecule ICAM-5 using experimental autoimmune encephalomyelitis (EAE), an animal model of MS. ICAM-5 knockout mice show a more severe EAE disease course in the chronic phase indicating a neuroprotective function of ICAM-5 in progressive neurodegeneration. In agreement with the predominant CNS-specific function of ICAM-5, lymphocyte function-associated antigen 1 (LFA-1)/ICAM-1 contact between antigen-presenting cells and T helper (Th)17 cells in EAE is not affected by ICAM-5. Strikingly, intrathecal application of the shed soluble form, sICAM-5, ameliorates EAE disease symptoms and thus might serve locally as an endogenous neuronal defense mechanism which is activated upon neuroinflammation in the CNS. In humans, cerebrospinal fluid from patients suffering from progressive forms of MS shows decreased sICAM-5 levels, suggesting a lack of this endogenous protective pathway in these patient groups. Overall, our study points toward a novel role of ICAM-5 in CNS autoinflammation in progressive EAE/MS.

## Introduction

Multiple sclerosis (MS) is an inflammatory neurodegenerative disease which is characterized by T cells infiltrating the central nervous system (CNS), thereby initiating autoimmune neuroinflammation (1–3). Subsequent to destabilization of the blood-brain barrier (BBB), proinflammatory cytokines are secreted by lymphocytes (4) thus orchestrating a proinflammatory environment via recruitment of other immune cells into the CNS (5). Throughout the pathogenesis of MS or in its animal model experimental autoimmune encephalomyelitis (EAE), adhesion molecules are not only involved in the migration process of lymphocytes into the CNS parenchyma via the BBB (6–8), they also contribute to reactivation of these immune cells via antigen-presenting cells (APCs). Recently, adhesion molecules have been shown to be involved in direct T cell interactions with neurons *in vivo*, thus inducing cell-cell contact-mediated neuronal calcium elevations (6, 9, 10).

Adhesion molecules play an important role for the regulation of peripheral immune processes. One prominent example is the intercellular adhesion molecule 1 (ICAM-1), which is typically expressed on endothelial and immune cells, and which can be induced by inflammatory cytokines such as interleukin-1 (IL-1) and tumor necrosis factor (TNF). Upon activation, leukocytes can bind to endothelial cells via lymphocyte function-associated antigen 1 (LFA-1)/ ICAM-1 and transmigrate into CNS tissue (11). In contrast, the related CNS-specific intercellular adhesion molecule 5 (ICAM-5, also called telencephalin (TLN) was first described as a regulator for brain development and the formation of synapses and is exclusively expressed on neurons in the telencephalon (12). A putative role of ICAM-5 in neuroinflammation is of high interest as pharmacological targeting might specifically address T cell-neuron contacts leading to T cell-mediated neuronal cell death. The binding partner of ICAM-5 is the β2-integrin LFA-1, which is expressed by invading leukocytes. LFA-1/ICAM-5 binding has therefore been proposed as a possible target to investigate neuroinflammatory processes in the CNS (13, 14). Additionally, ICAM-5 can be cleaved by matrix metalloproteinase (MMP)-2 and 9 from the neuronal surface (15) and the soluble form (sICAM-5) has been proposed to act as an inhibitor of ICAM-1–LFA-1 interactions between CD4^+^ T cells and APCs (16); the involvement of pathogenic Th17 cells has not been addressed so far. Interestingly, encephalitis patients show elevated concentrations of sICAM-5 in the cerebrospinal fluid (CSF); however its pathological relevance is still unclear (17).

**Figure 1:**
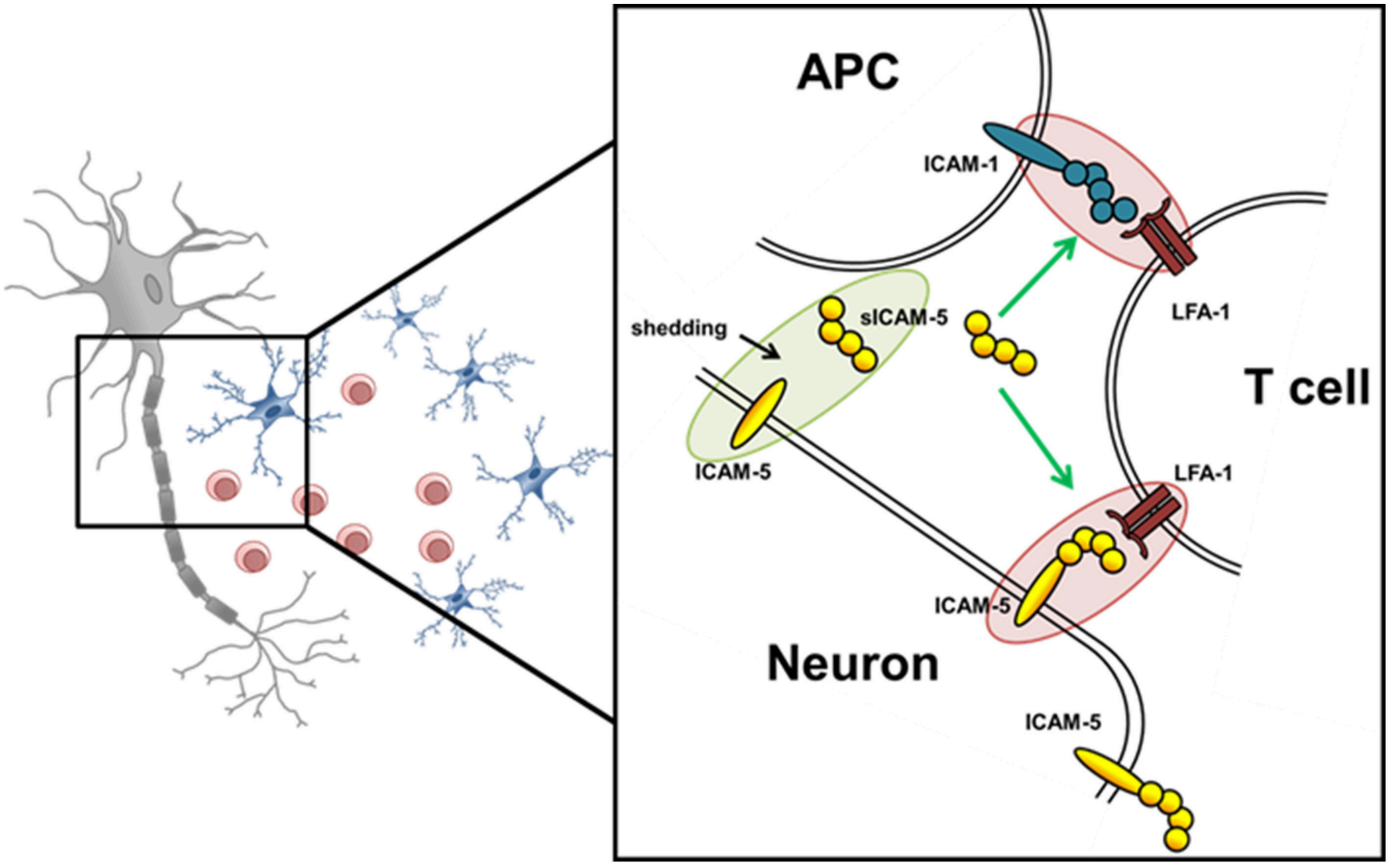
Schematic overview of potential ICAM-5 dependent T cell—neuron and T cell—APC interactions in the CNS. Schematic illustration of the interplay of neurons, T cells, and APCs in the CNS. The adhesion molecule ICAM-5 (yellow) is exclusively expressed by neurons and can be cleaved by MMP-2 and 9 from the neuronal surface (also called shedding). The soluble form (sICAM-5) has been proposed to act as an inhibitor of ICAM1–LFA-1 interactions between T cells and APCs and T cells and neurons therefore displaying a protective function. Green highlights and arrows represent anti-inflammatory/ protective function and red highlights represent proinflammatory properties.

Here, we examined the role of ICAM-5 as a direct binding partner between neurons and T helper 17 (Th17) cells in MS pathology, and investigated whether the interaction of its soluble form, sICAM-5, influences the interactions between Th17 cells and APCs thereby serving as an immunosuppressive molecule (Figure 1). The functional expression of ICAM-5 in the CNS and secretion levels of protective sICAM-5 proteins in the CSF of MS patients was assessed to provide insights into the relevance of ICAM-5 for the human system. Our results contribute to a deeper understanding of immune cell-driven neuronal injury which may influence future therapeutic approaches.

## Material and Methods

### Experimental Autoimmune Encephalomyelitis

ICAM-5 knockout (KO) mice were originally provided by the Gahmberg laboratory, University of Helsinki (Finland); C57BL/6 mice were purchased from Janvier laboratory (France). All animal experiments were approved by local authorities and conducted according to the German Animal Protection Law. Active EAE in C57BL/6, ICAM5-KO mice and littermates was induced using the Hooke Kit™ MOG_35−55_/CFA Emulsion + PTX following the manufacturer's protocol. Thus, mice were actively immunized by subcutaneous injection of 200 μg emulsion of MOG_35−55_ in complete Freund's adjuvant (CFA), followed by the administration of two intraperitoneal doses of 400 ng of pertussis toxin (PTX) in PBS, at the time of immunization and 48 h later. Clinical signs of EAE were monitored daily and translated into clinical scores as follows: 0, no detectable signs of EAE; 0.5, tail weakness; 1, complete tail paralysis; 1.5, impaired righting reflex; 2, partial hind limb paralysis; 2.5, unilateral complete hind limb paralysis; 3, complete bilateral hind limb paralysis; 3.5, complete hind limb paralysis and partial forelimb paralysis; 4, total paralysis of forelimbs and hind limbs; and 5, death. EAE mice were treated with steroid pulses of 16 μg/g Urbason (Methylprednisolone) given via intraperitoneal injections on five consecutive days as soon as a clinical score of 2 was reached, supporting the development of a chronic progressive EAE disease course in this model. The 16 μg/g dose was based on the clinical human dose of 1,000 mg per day and a median weight of 63 kg (human) vs. 20 g (mouse).

### Intrathecal Injection of ICAM-5 D1-2-Fc by Lumbar Injection

Recombinant mouse ICAM-5 Fc chimera protein (ICAM-5 D1-2-Fc, R&D Systems, USA) was delivered intrathecally by lumbar puncture in awake mice as described elsewhere (18, 19). In short, the mouse was grasped at the iliac crest, so that both hind legs move outward and downward. Then the Hamilton syringe/needle was inserted at about a 45° angle between the L5 and L6 spinous process and 5 μl injection solution was applied. A reflexive flick of the tail indicated puncture of the dura. After reaching a clinical score of 2, EAE mice received 0.2 μg of ICAM-5 D1-2-Fc in this manner seven times at 48-h intervals to induce an ICAM-5 D1-2-Fc concentration in the CSF of 5 μg/ml. The control group was treated with IgG peptide.

### Murine Cell Culture

Naïve CD4^+^CD62L^+^ cells were isolated and MACS-sorted from spleens and lymph nodes of B6.2D2 and B6.RFP.2D2 mice (6–10 weeks old) with a purity of >95% of total cells. Murine Th17 cell differentiation was achieved by the addition of 2 μg/ml anti-CD3, 3 ng/ml TGF-β, 20 ng/ml mrIL-6, 20 ng/ml IL-23, 10 μg/ml anti-IL-4, and 10 μg/ml anti-IFNγ. Irradiated APCs were used for initial stimulation of the T lymphocytes in a ratio 1:10. Cells were kept in cell culture medium and were split with 50 U/ml IL-2 and 10 ng/ml IL-23. Cells that produced >30% of IL-17 were used for *in vitro* assays. Cytokine production was assessed using intracellular cytokine staining following standard protocols (CD4-PeCy7 (clone: RM4-5, BD Bioscience), IL-17A-AF647 (clone: 17B7, Affimetrix), IFNγ-Horizon (clone: XMG1.2, BD Bioscience), TNFα-AF700 (clone: MP6-XT22BD, Bioscience)). Surface stainings were performed with CD44-AF700 (clone: IM7, eBioscience), CD49d-FITC (clone: R1-2, eBioscience), CD54-APC (clone: YN1/1.7.4, Biolegend) and MHCII-PerCP (clone: AF6-120.1, BD), CD62L-APC (clone: MEL-14, BD Biosciences), CD69-PE (clone: H1-2F3, BD Biosciences), CD40L-PECy7 (clone: MR1, Biolegend), and CD25-PECy7 (clone: PC61, BD Biosciences). ICAM-5 treatment was performed by adding 10 μg/ml ICAM-5 D1-2-Fc (R&D Systems, USA) either during the whole time of Th17 cell differentiation or after restimulation of differentiated Th17 cells.

### Proliferation Assay

Carboxyfluorescein succinimidyl ester (CFSE) proliferation assay was performed by incubating naïve T cells in mouse-medium at 37°C for 30 min, then cells were washed twice with pre-warmed RPMI+1% HEPES (RH) and then dissolved in CFSE in pre-warmed RH at a concentration of 2.5 μM. After quenching the stained cells with cold mouse-medium, cells were incubated for at least 72 h and evaluated by flow cytometric measurements.

### Cortical Neuronal Cell Culture

For neuronal cultures, p0-p1 pups were beheaded and brains were removed from the skull. The brains were prepared in ice-cold Hank's balanced salt solution (HBSS). The olfactory bulbs and the meninges were removed from the cortex. The hippocampus was stripped from the cortex and all cortices were collected in ice-cold HBSS. Cortices from up to three animals were pooled into one falcon tube. The tissue was washed once with ice-cold HBSS and digested in HBSS with 1% DNAse and 0.5% trypsin for 20 min at 37°C. For homogenizing, tissue was sucked through two small glass pipettes and finally poured over a 70 μm cell sieve. 500,000−750,000 cells were seeded in each well of the 6-well plate in plating medium. After 3 h cells were washed with neuro basal medium. The cultures were washed every 2–3 days. Neuronal cultures were inflamed between d7 and d8 with LPS (10 μg/ml), IFNγ (100 ng/ml) or splenocyte supernatant. Cultures were harvested 24 h later, between d8 and d9. Cells were harvested with 3% trypsin for 5 min at 37°C, washed from the plates and collected on ice. The cells were centrifuged for 5 min at 550 g at 4°C and washed once with ice-cold phosphate-buffered saline (PBS). Pellets for mRNA analysis were frozen at −80°C.

### Quantitative Real-Time PCR

For analysis of ICAM-5 and MMP-9 expression, RNA was isolated using the RNeasy Mini Kit (Quiagen) according to the manufacturer's protocol; quality and integrity of total RNA preparation was confirmed using a NanoDropTM 2000c Spectrophotometer (Thermo Scientific). Complementary DNA (cDNA) synthesis was performed by reverse transcription of total RNA using the SuperScriptIII First Strand Synthesis System and random hexamer primers (Invitrogen) following the manufacturer's instructions. Amplification primers for real-time PCR analysis were designed using Beacon Designer 8 Software (PREMIER Biosoft International) according to the manufacturer's guidelines and subsequently tested for amplification efficiency and specificity. Real-time PCR was performed using iQ SYBR Green supermix (BioRad Laboratories) in an CFX Connect TM Real Time Detection System (BioRad). Primer sequence as given: ICAM-5 (forward: CGT ATG TAT TGT TCG CTC TC; reverse: TTA TTG AAG GGA ATG GGT AGA) and MMP-9 (forward: AAG TCT CAG AAG GTG GAT; reverse: AAT AGG CTT TGT CTT GGTA). Relative changes in gene expression were determined using the Ct method (20) with β-actin as the reference gene.

### Immunohistochemistry

Immunohistochemistry was performed with neuronal cortical cultures of B6.2D2 mice. Neurons were stained for ICAM-5 (TubIII, Covance), NeuN (Millipore, Billerica), MAP2 (HM-2, Sigma), and Tuj1 (Covance); cell nuclei were stained with DAPI (Invitrogen). Pictures were obtained using a confocal laser scanning microscope (Leica TCS-SP8; Leica Microsystems Heidelberg, Mannheim, Germany).

### Human ICAM-5 ELISA

CSF samples were collected at the time of first diagnosis from 48 patients fulfilling the revised McDonald criteria for MS (21) which included 17 with relapsing-remitting MS (RRMS, age: 38.6 ± 1 years, range: 21–78 years, EDSS: 1.7 ± 0.1, range: 0–3.5, disease duration: 15.6 ± 1.7 months, range: 0–84 months), 19 with primary progressive MS (PPMS, age 56.7 ± 0.5 years, range: 42–70 years, EDSS 3.7 ± 0.1, range: 1–6.5, disease duration 45.2 ± 2.5 months, range: 1–156 months), and 12 with secondary progressive MS (SPMS, age 48.5 ± 0.7 years, range: 34–67 years, EDSS 4.5 ± 0.2, range: 2–7.5, disease duration 99.5 ± 8.3 months, range: 18–300 months). Samples from 35 patients with non-inflammatory neurological disease (NIND, mean (± SEM) age: 42.3 ± 0.5 years, range: 17–79 years) served as controls. CSF was collected and kept frozen at −80°C until assayed. ICAM-5 levels were measured using a human ICAM-5 ELISA kit (DY1950-05, R&D Systems) with an ancillary reagent pack according to the manufacturers' protocol (DY008, R&D Systems). Fluorescence was determined using an Infinite M200 pro reader (Tecan). This study was approved by the local ethical committee (Landesaerztekammer Rheinland-Pfalz) and performed according to the Helsinki Declaration; all patients provided written informed consent.

### Histology

Cryosections of EAE mouse brains were stained for hematoxylin and eosin (HE), luxol fast blue stain with periodic acid- Schiff's (LFB-PAS), amyloid precursor protein (APP), and neurofilament (NF). Pictures were obtained using an Olympus microscope equipped with a cellSense camera and analyzed with ImageJ software.

### NfL Measurements

Briefly, blood or CSF samples were spun at 2,000 g at room temperature for 10 min within 2 h after withdrawal and stored in polypropylene tubes at −80°C. Neurofilament levels in serum and CSF were measured by SiMoA HD-1 (Quanterix, USA) using the NF-Light Advantage Kit (Quanterix) according to manufacturer's instructions. Samples were measured in duplicates; the intra-assay coefficient of variation (CV) of all samples was below 20%. sNfL measurements were performed in a blinded fashion without information about clinical data.

### Statistics

Statistical analyses were performed with Graphpad Prism 6. We used a K-S test for normality, followed by Mann-Whitney test, unpaired two-tailed Student's *t*-test or one-way ANOVA followed by Tukey's multiple comparison test, as appropriate. Data are presented as the mean ± standard error of the mean (SEM) unless otherwise indicated. *P*-values < 0.05 were considered statistically significant.

## Results

### sICAM-5 Has No Impact on Th17 Cell—APC Interaction

**Figure 2:**
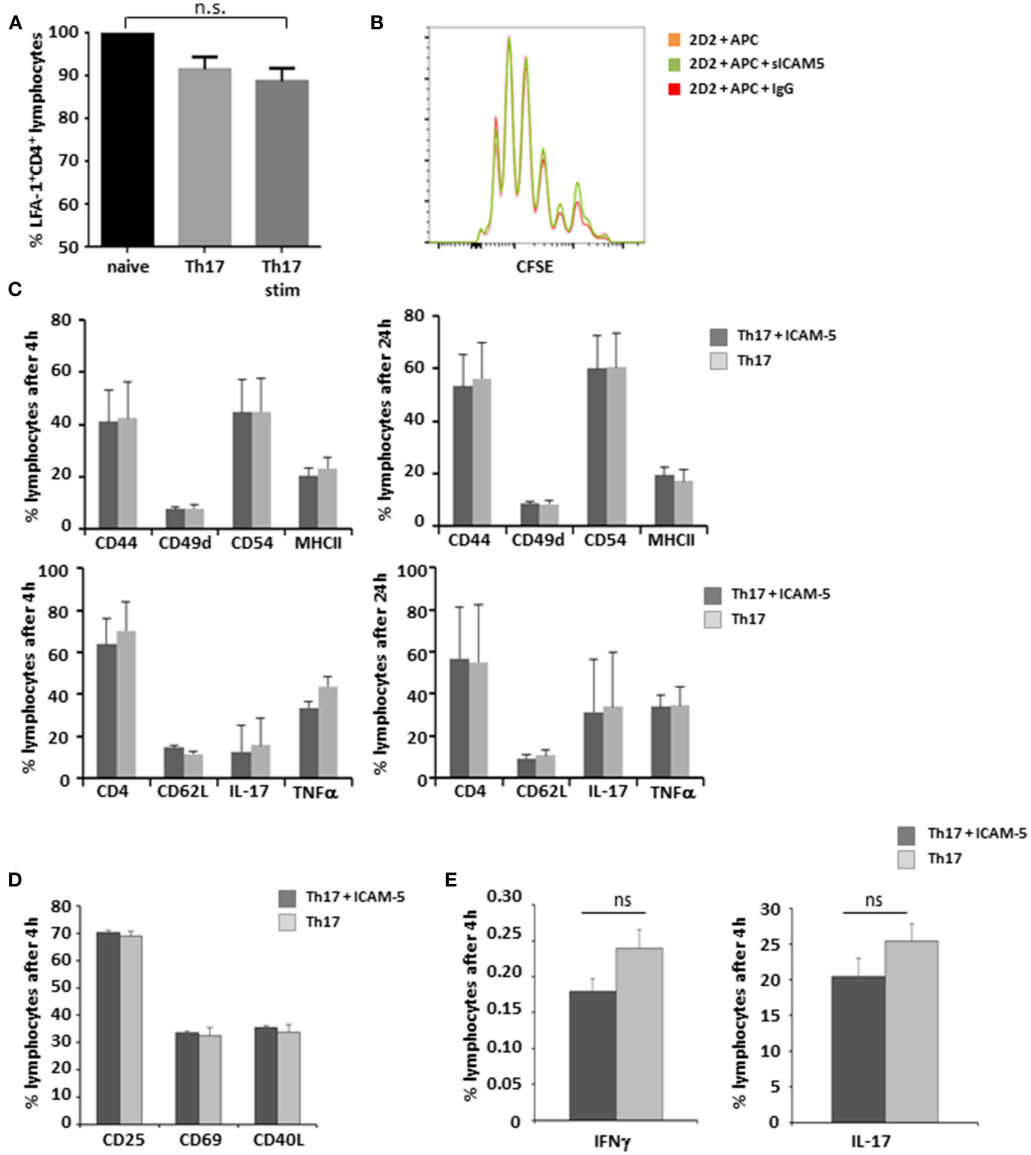
sICAM-5 does not affect Th17—APC interactions. **(A)** FACS staining for LFA-1 of naïve T-cells, Th17 cells, and Th17 cells after 24 h of stimulation with anti-CD3/CD28 (*n* = 3 for each condition). **(B)** Representative depiction of CFSE stainings of naïve 2d2 T cells cocultured with APCs and sICAM-5 (10 μg/ml) or IgG (10 μg/ml) for 72 h. **(C)** FACS staining of Th17 cells stimulated for 4 and 24 h with anti-CD3/CD28 and with or without ICAM-5 treatment (10 μg/ml). **(D,E)** Naïve T cells were differentiated into Th17 cells in the presence of or without sICAM-5 treatment (10 μg/ml) and stained for extracellular **(D)** and intracellular **(E)** markers. Data shown are mean ± SEM. **(A)** One-way ANOVA followed by Tukey's multiple comparison test, n.s. no significance; **(C–E)** Student's *t*-test.

LFA-1 is a central binding partner of both ICAM-5 and ICAM-1 (16). Since Th17 cells play a major role in the immunopathology of both EAE and MS (22, 23), we first aimed to elucidate the influence of sICAM-5 on the interactions of this pathogenic T cell type with APCs. There were no significant differences in the expression levels of LFA-1 on either cell type (Figure 2A) indicating that T-cell differentiation and stimulation are independent of LFA-1 expression. Next, we analyzed the influence of sICAM-5 on the APC-dependent proliferation of Th17 cells and found no differences in the proliferation between the sICAM-5-treated T cells and control conditions (Figure 2B). Furthermore, we did not see any significant differences in the expression of CD44, CD49d, CD54, MHC class II, CD62L, IL-17, or TNFα after 4 or 24 h of stimulation with CD3/CD28 and sICAM5 on differentiated Th17 cells (Figure 2C). We moreover assessed the differentiation of naïve T cells into Th17 cells in the presence of sICAM-5 and did not find significant differences in the expression of surface markers CD69, CD25, CD40L, or intracellular cytokine levels for IL-17 and IFNγ (Figures 2D,E). Overall, these results indicate that sICAM-5 does not directly alter the differentiation and function of murine Th17 cells themselves. Both species differences between mouse and human and our focus on the Th17 cell subtype of T cells might explain a different outcome compared to previous reports on human naïve CD4^+^ T cells (24).

### Absence of ICAM-5 Worsens Disease Progression in Active EAE Which Can be Reversed by sICAM-5 Application

**Figure 3:**
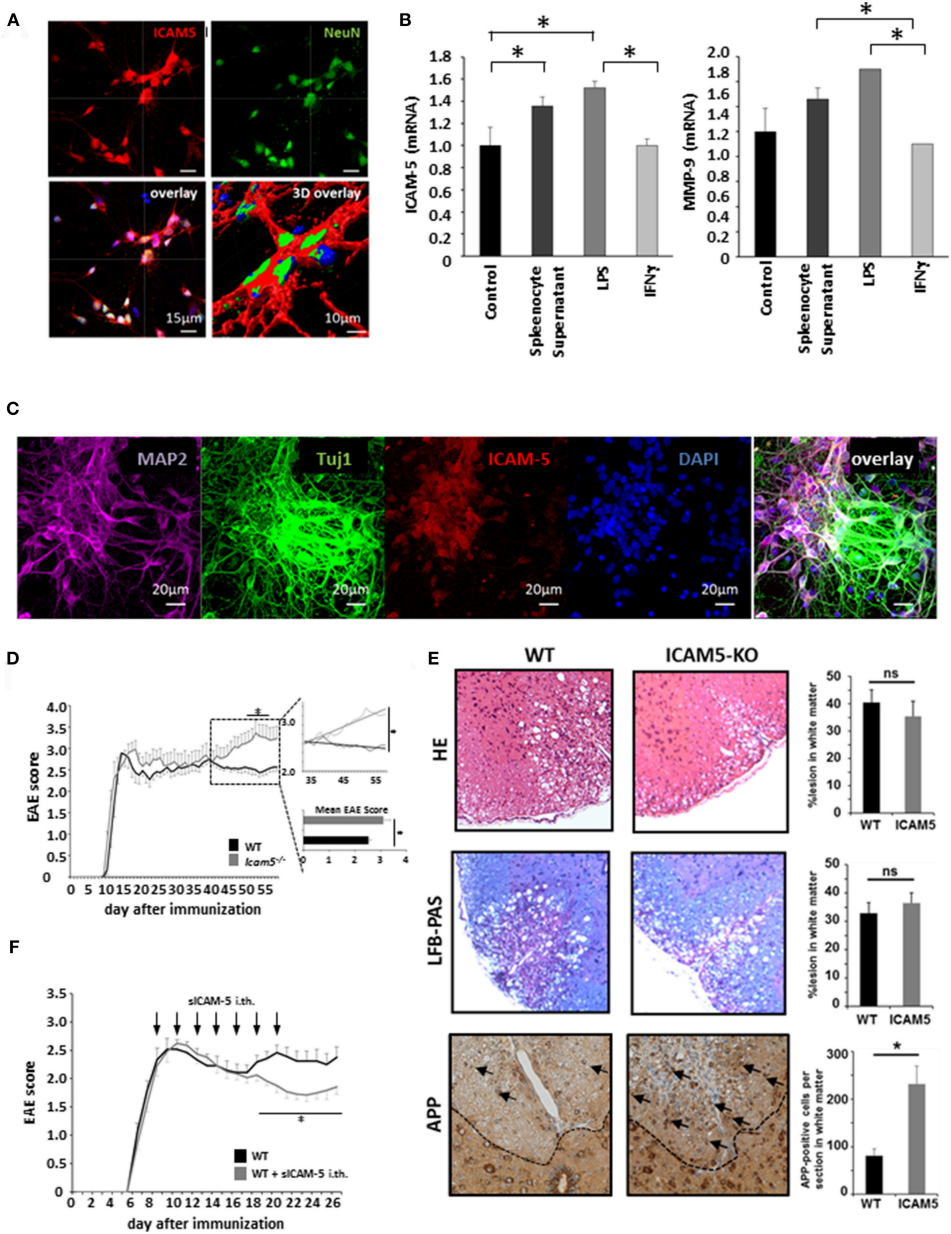
Absence of ICAM-5 worsens disease progression which can be reversed by application of sICAM-5. **(A)** Immunohistochemical staining of ICAM-5-AF647 on cortical neurons (scale bar = 15 μm, 3D overlay scale bar = 10 μm). Co-staining was performed with NeuN-AF488 and DAPI (blue). **(B)** mRNA analysis of ICAM-5 and MMP-9 were performed with cortical neurons after stimulation with LPS, IFNγ, and splenocyte supernatant (*n* = 6 for each condition). Unstimulated cortical neurons served as a control. **(C)** Immunohistochemical staining of MAP2- AF647, Tuj1-FITC, ICAM-5-AF568, and DAPI on cortical neurons (scale bar = 20 μm. **(D)** EAE was induced actively in ICAM-5-KO mice and WT littermates via the injection of MOG_35−55_ (two independent EAEs, untreated; WT *n* = 12, KO *n* = 12). The dotted box on the plot provides a closer look at the chronic phase of the EAE but does not agree with the x axis of the upper plot. The lower plot shows only the mean disease score of the period from day 40 after immunization. **(E)** EAE lesions were stained from wt (*n* = 9) and ICAM5 KO mice (*n* = 6) for inflammation (HE staining), demyelination (LFB-PAS), and axonal loss (APP) and were quantified accordingly. **(F)** EAE was induced actively in B6 mice via the injection of MOG_35−55_ and mice were treated with Methylprednisolone for 5 days as soon as the mice reached a clinical score of 2. Additionally, once animals reached a clinical score of 2, 0.2 μg ICAM-5 D1-2 Fc or human IgG was applied via intrathecal (i.th) injection by lumbar puncture seven times on every second day (WT *n* = 7, KO *n* = 7). Data shown are mean ± SEM. **(C, D)** Mann-Whitney *U*-test; *p < 0.05.

Despite its potential to regulate T cell restimulation as well as function as an adhesion molecule for lymphocytes *in vitro*, the relevance of ICAM-5 in neuroinflammation has not been investigated *in vivo*. To this end, we first cultured dissociated cortical neurons and stained them for NeuN and ICAM-5 (Figure 3A). Indeed, ICAM-5 is highly expressed by neurons. We found a further upregulation of both ICAM-5 and the matrix metalloproteinase 9 (MMP-9), a protein able to cleave ICAM-5 from the neuronal surface, after inflammatory stimulation with LPS and splenocyte supernatant, but not with IFNγ (Figure 3B). Additional neuronal markers MAP-2 or beta-III tubulin (Tuj1) were used to stain cortical neurons and to validate that ICAM-5 is preferentially expressed on soma and dendrites (Figure 3C) (16). Next, we induced EAE in mice deficient for ICAM-5 and significant differences were observed between the two groups exclusively in the chronic phase of disease (Figure 3D). Taking into account possible pro- and anti-inflammatory effects of ICAM-5 in the context of EAE (see also Figure 1), this indicates that the biologically relevant function of ICAM-5 *in vivo* is to serve as an endogenous neuroprotective pathway in chronic neurodegeneration, most likely via sICAM-5-mediated effects. Further, histopathological stainings were performed for inflammation (HE staining), demyelination (LFB-PAS), and axonal loss (APP) in the lesions of these EAE mice. Neither inflammation nor demyelination was reduced in ICAM5-KO mice, though axonal loss was significantly elevated (Figure 3E), arguing in favor of predominating axonal pathology. To examine the impact of sICAM-5 in ongoing neuroinflammation, we applied sICAM-5 locally via intrathecal injection by lumbar puncture during EAE. Indeed, sICAM-5-treated mice displayed a more pronounced recovery after the peak of disease as compared to the control group which was injected with a control IgG peptide (Figure 3F).

### Patients Suffering From Progressive Forms of MS Show Low Levels of sICAM-5 in CSF

**Figure 4:**
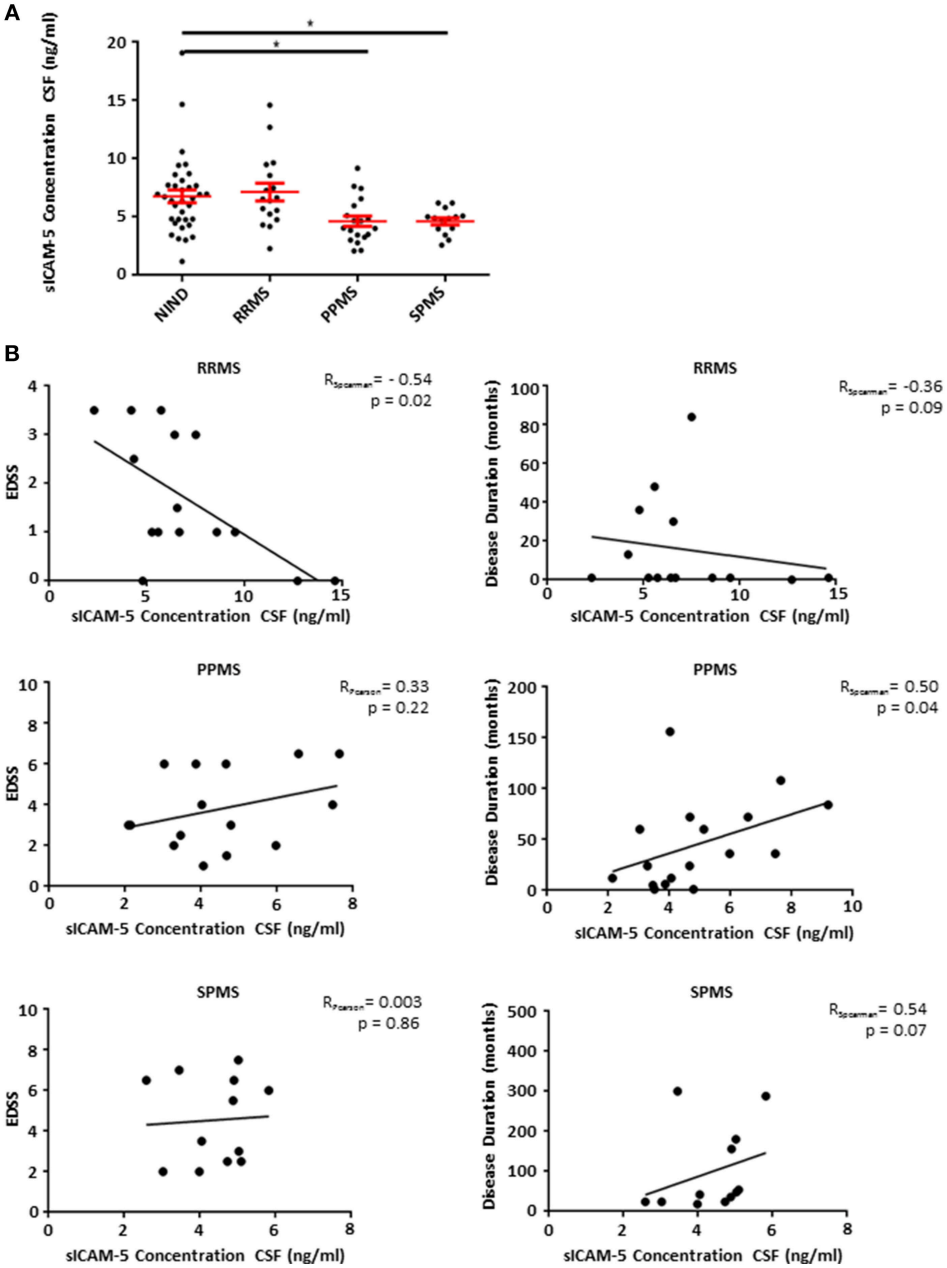
Patients suffering from progressive forms of MS show lower levels of sICAM-5 in CSF. **(A)** ICAM-5 concentration in ng/ml in the cerebrospinal fluid of NIND patients (NIND, *n* = 35), RRMS (*n* = 17), PPMS (*n* = 19), and SPMS (*n* = 12) patients. Data shown are mean ± SEM. One-way ANOVA followed by Tukey's multiple comparison test, *p < 0.05. **(B)** Correlation between EDSS or disease duration in months and ICAM-5 concentration in CSF (ng/ml). Data was tested for normality using Shapiro-Wilk normality test and Pearson's or Spearman's r and *p*-values were determined accordingly.

Based on our EAE findings indicating an endogenous protective role of sICAM-5, we assessed the role of sICAM-5 in the CSF of patients with MS. CSF samples from RRMS, SPMS, and PPMS patients as well as from a control group (NIND) with normal CSF findings were analyzed using a human ICAM-5 ELISA. While RRMS and control patients showed comparable levels of sICAM-5, sICAM-5 levels in PPMS, and SPMS patients were significantly lower (Figure 4A). This suggests that a reduction in ICAM-5 shedding in humans may play a role specifically in chronic forms of MS. This might either indicate a loss-of-function of an endogenous protective pathway driving neurodegeneration or a secondary effect due to a higher neuronal loss in progressive phases of MS and therefore a lower availability of sICAM-5. We moreover correlated sICAM-5 concentration in CSF with the EDSS score and disease duration (Figure 4B) for each MS patient subtype. In RRMS, we found a moderate correlation between EDSS and sICAM-5 concentration in the CSF with higher EDSS scores being associated with a lower concentration of sICAM-5 in the CSF. In SPMS and PPMS, we found a correlation between disease duration and sICAM-5 concentration with higher sICAM-5 concentrations correlating with longer disease duration. We found no correlation between age and sICAM-5 levels (Supplementary Figure 1).

### No Significant Correlation Between Neurofilament Light Chain Levels and sICAM-5

**Figure 5:**
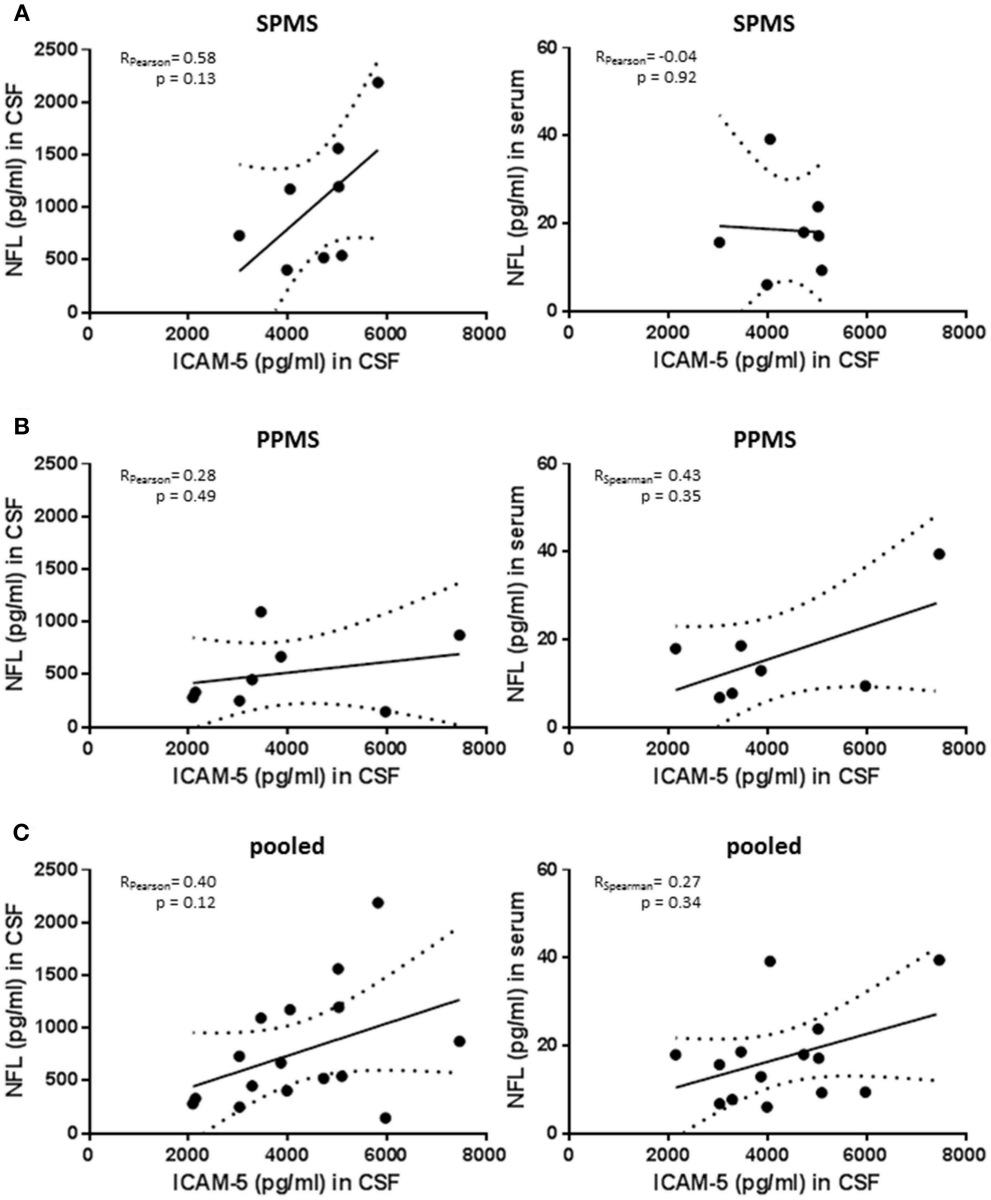
No significant correlation between neurofilament light chain levels and sICAM-5. **(A)** ICAM-5 was correlated with NfL concentrations (pg/ml) in the cerebrospinal fluid and the serum of SPMS patients (*n* = 7–8). **(B)** ICAM-5 was correlated to NfL concentrations (pg/ml) in the cerebrospinal fluid and the serum of PPMS patients (*n* = 7–8). **(C)** ICAM-5 was correlated to NfL concentrations (pg/ml) in the cerebrospinal fluid and the serum of pooled SPMS and PPMS patients (*n* = 14–16). For all figures: Data was tested for normality using Shapiro-Wilk normality test and Pearson's or Spearman's r and *p*-values was determined accordingly.

Neurofilament light chain (NfL) is emerging as a biomarker assessing neuroaxonal damage in patients with multiple sclerosis while knowledge about its relevance in patients with progressive MS is still limited. We measured NfL serum and CSF concentration in the PPMS and SPMS group and correlated these values with ICAM-5 levels (Figures 5A–C). Of note, although reflecting neurodegeneration in MS patients NfL is sensitive to almost every type of neuronal damage including inflammatory causes (25). SPMS patients showed higher NfL levels than observed in PPMS patients, confirming previous results derived from larger cohorts [SPMS *n* = 1452 and PPMS *n* = 378; retrospective analysis of INFORMS and EXPAND trials presented by Kuhle et al. (26) ECTRIMS 2018] and CSF levels were highly correlated with serum concentration (Spearman rho = 0.684; *p* = 0.007). In the SPMS subgroup, CSF NfL significantly correlated with the EDSS scores and disease duration (Spearman rho = 0.843, *p* = 0.009 and Spearman rho = 0.838, *p* = 0.009, respectively). Notably, the correlation between NfL and EDSS score is still depicted in the literature by contrary results (27–30). We observed only trends regarding the association between NfL and ICAM-5 in this study but NfL values are drastically different between individuals. There is therefore a need for large cohorts to show significant cross-sectional differences between groups, and we should be aware of our small study population size when interpreting these results. In addition, a more powerful interpretation of sNfL might be achieved by using the individual rate of change of NfL rather than cross-sectional absolute NfL levels (31). The comparison of NfL and ICAM-5 in this study is aggravated by the fact that ICAM-5 is not only released in the extracellular space upon cell damage but, as we showed, its expression is changed upon different stimuli and its concentration in CSF depends on MMP9 activity. In conclusion, combining ICAM-5 with NfL levels in future studies will certainly help to decipher the role of ICAM-5 in neurodegeneration in more detail, but in this study is limited by the small cohort size and different release dynamics.

## Discussion

In MS, T cells directly interact with neurons *in vivo* inducing neuronal calcium elevations thus leading to neuronal damage. Here, we investigated the role of ICAM-5 in mediating T cell-neuron contacts. ICAM-5 is exclusively expressed on neurons where it can serve as a binding partner to LFA-1 on invading leukocytes. Soluble ICAM-5 might act as an endogenous regulator of neuronal damage as it has anti-inflammatory effects after binding to LFA-1. Thus, neuronally released sICAM5 can modulate immune cell-neuron as well as T cell-APC interactions. Indeed, ICAM-5 expression was detected on neuronal cell cultures *in vitro*, but not on other cell types. Induction of MOG_35−55_ peptide-induced EAE in wildtype (WT) and ICAM-5^−/−^ mice led to an unchanged phenotype in the inflammatory phase, followed by a significant deterioration in the progressive phase. For the interpretation of results from the ICAM-5^−/−^ mouse, it has to be kept in mind that the full knockout of ICAM-5 might have both beneficial effects (via deletion of T cell-neuron binding capacity) and detrimental effects (since the beneficial effects of sICAM-5 would also be abrogated). The EAE phenotype underlines that the protective effect of sICAM-5 is of major biological importance, especially in the chronic phase of EAE. To further address the therapeutic potential of ICAM-5 modulation, sICAM-5 was applied intrathecally into WT mice, to address only the CNS milieu, leading to a subsequent amelioration of clinical disease symptoms. Although it cannot be excluded that the recombinant ICAM-5 chimera used in these experiments may behave differently to the endogenously shed sICAM-5, the improvement in EAE disease symptoms suggests that sICAM-5 serves locally as an endogenous neuronal defense mechanism which might be activated upon neuroinflammation in the CNS.

Comparing the so far known effects of ICAM-5 to its related molecule ICAM-1 is interesting since ICAM-1 mostly localizes to glia and endothelial cells while ICAM-5 is restricted to telencephalic neurons (24). ICAM-1 was shown to have proinflammatory effects on macrophages (32) but to also influence T cell receptor signaling by slowing the actin flows in primary CD4^+^ T cells especially with regard to T cell interactions with dendritic cells (33). Contrary to ICAM-1, ICAM-5 was shown to have a rather immune-suppressive function in T cells (24). Neurons which were stimulated with LPS or splenocyte supernatant upregulated ICAM-5 and MMP-9 mimicking the situation of inflammation. Since we were able to exclude a direct effect of ICAM-5 on Th17 cell pathology we hypothesize that ICAM-5 might also play an important role in the interaction of neurons with APCs such as dendritic cells and microglia in EAE. Interestingly, LFA-1 is highly expressed by microglia in chronic active lesions (34) and can be bound by ICAM-5, again indicating a potential neuroprotective role of ICAM-5 especially in the chronic phase of MS.

Furthermore, it is known that ICAM-5 regulates neuronal development in several ways and is highly expressed on the dendrites and soma of neurons (35) and during the initial contact between the pre- and the postsynaptic terminals, ICAM-5 stabilizes postsynaptic connections by binding to β1 integrins (36). β1 integrins are also expressed by APCs such as microglia (37). Interestingly, there is direct proof that endogenous ICAM-5 from neurons is bound by microglia and induces clustering and weak adhesion of microglia (38). Additionally, ICAM-5 downregulates phagocytosis in unchallenged microglia and promotes IL-10 production in LPS-stimulated microglia (38) thus further suggesting a potential role of ICAM-5 in the regulation of microglia in the chronic phase of disease. These microglia-related ICAM-5 pathways merit further investigation.

Importantly, CSF from patients suffering from a progressive form of MS show decreased ICAM-5 levels. This is in accordance with our murine data showing a neuroprotective effect in the chronic phase of the disease, underlining the importance of ICAM-5 in the human system. Moreover, ICAM-5 concentrations in the CSF correlate with disease duration in patients with progressive forms of MS. It has previously been shown that sICAM-5 is cleaved and released into the CSF also in other neurological diseases such as acute encephalitis (17). Although our study could not classify the exact pathophysiological role of sICAM-5 release into the CSF in progressive MS patients, interesting possibilities are raised. ICAM-5 has been discussed as a marker of neuronal death in traumatic brain injury (39). Increased levels of sICAM-5 in the CSF could therefore be caused by an increase in neuronal death and thereby caused release of ICAM-5. As there is a link between chronic disease, microglia activation and neuronal ICAM-5 (38), the positive correlation between sICAM-5 and disease duration could also point toward other involved pathways which are not addressed in the current study. Interestingly, although there was no significant difference in ICAM-5 levels between NIND and RRMS patients, there was a strong negative correlation between EDSS and ICAM-5 concentration in the CSF of RRMS patients, which supports our hypothesis.

## Data Availability

The datasets generated for this study are available on request to the corresponding author.

## Author Contributions

KB, JL, and SB conceived the study. KB, JL, RG, FS, BW, TR, and SM performed experiments and analyzed data. KB, JL, FZ, and SB wrote and edited the manuscript. This work contains part of the doctoral thesis of JL.

### Conflict of Interest Statement

The authors declare that the research was conducted in the absence of any commercial or financial relationships that could be construed as a potential conflict of interest.

## References

[1] Cua DJ Sherlock J Chen Y Murphy CA Joyce B Seymour B. Interleukin-23 rather than interleukin-12 is the critical cytokine for autoimmune inflammation of the brain. Nature. (2003) 421:744–8. 10.1038/nature01355 12610626

[2] Langrish CL Chen Y Blumenschein WM Mattson J Basham B Sedgwick JD. IL-23 drives a pathogenic T cell population that induces autoimmune inflammation. J Exp Med. (2005) 201:233–40. 10.1084/jem.20041257 15657292 PMC2212798

[3] Komiyama Y Nakae S Matsuki T Nambu A Ishigame H Kakuta S. IL-17 plays an important role in the development of experimental autoimmune encephalomyelitis. J Immunol. (2006) 177:566–73. 16785554 10.4049/jimmunol.177.1.566

[4] Bittner S Wiendl H. Neuroimmunotherapies Targeting T Cells: from pathophysiology to therapeutic applications. Neurotherapeutics. (2016) 13:4–19. 10.1007/s13311-015-0405-3 26563391 PMC4720668

[5] Ellwardt E Walsh JT Kipnis J Zipp F. Understanding the Role of T Cells in CNS Homeostasis. Trends Immunol. (2016) 37:154–65. 10.1016/j.it.2015.12.008 26775912

[6] Larochelle C Alvarez JI Prat A. How do immune cells overcome the blood-brain barrier in multiple sclerosis? FEBS Lett. (2011) 585:3770–80. 10.1016/j.febslet.2011.04.066 21550344

[7] Engelhardt B. Cluster: barriers of the central nervous system. Acta Neuropathol. (2018) 135:307–10. 10.1007/s00401-018-1816-0 29397420

[8] Lopes Pinheiro MA Kooij G Mizee MR Kamermans A Enzmann G Lyck R Immune cell trafficking across the barriers of the central nervous system in multiple sclerosis and stroke. Biochim Biophys Acta. (2016) 1862:461–71. 10.1016/j.bbadis.2015.10.018 26527183

[9] Paterka M Siffrin V Voss JO Werr J Hoppmann N Gollan R. Gatekeeper role of brain antigen-presenting CD11c+ cells in neuroinflammation. Embo J. (2016) 35:89–101. 10.15252/embj.201591488 26612827 PMC4718005

[10] Siffrin V Radbruch H Glumm R Niesner R Paterka M Herz J. *In vivo* imaging of partially reversible th17 cell-induced neuronal dysfunction in the course of encephalomyelitis. Immunity. (2010) 33:424–36. 10.1016/j.immuni.2010.08.018 20870176

[11] Rothlein R Dustin ML Marlin SD Springer TA. A human intercellular adhesion molecule (ICAM-1) distinct from LFA-1. J Immunol. (1986) 137:1270–4. 3525675

[12] Mori K Fujita SC Watanabe Y Obata K Hayaishi O. Telencephalon-specific antigen identified by monoclonal antibody. Proc Natl Acad Sci USA. (1987) 84:3921–5. 3295872 10.1073/pnas.84.11.3921 PMC304988

[13] Tian L Yoshihara Y Mizuno T Mori K Gahmberg CG. The neuronal glycoprotein telencephalin is a cellular ligand for the CD11a/CD18 leukocyte integrin. J Immunol. (1997) 158:928–36. 8993013

[14] Tian L Kilgannon P Yoshihara Y Mori K Gallatin WM Carpen O. Binding of T lymphocytes to hippocampal neurons through ICAM-5 (telencephalin) and characterization of its interaction with the leukocyte integrin CD11a/CD18. Eur J Immunol. (2000) 30:810–8. 10.1002/1521-4141(200003)30:3<810::aid-immu810>3.0.co;2-x 10741396

[15] Tian L Stefanidakis M Ning L Van Lint P Nyman-Huttunen H Libert C. Activation of NMDA receptors promotes dendritic spine development through MMP-mediated ICAM-5 cleavage. J Cell Biol. (2007) 178:687–700. 10.1083/jcb.200612097 17682049 PMC2064474

[16] Gahmberg CG Tian L Ning L Nyman-Huttunen H. ICAM-5–a novel two-facetted adhesion molecule in the mammalian brain. Immunol Lett. (2008) 117:131–5. 10.1016/j.imlet.2008.02.004 18367254

[17] Lindsberg PJ Launes J Tian L Valimaa H Subramanian V Siren J. Release of soluble ICAM-5, a neuronal adhesion molecule, in acute encephalitis. Neurology. (2002) 58:446–51. 10.1212/WNL.58.3.446 11839847

[18] Lu R Schmidtko A. Direct intrathecal drug delivery in mice for detecting *in vivo* effects of cGMP on pain processing. Methods Mol Biol. (2013) 1020:215–21. 10.1007/978-1-62703-459-3_14 23709036

[19] Vulchanova L Schuster DJ Belur LR Riedl MS Podetz-Pedersen KM Kitto KF. Differential adeno-associated virus mediated gene transfer to sensory neurons following intrathecal delivery by direct lumbar puncture. Mol Pain. (2010) 6:31. 10.1186/1744-8069-6-31 20509925 PMC2900238

[20] Livak KJ Schmittgen TD. Analysis of relative gene expression data using real-time quantitative PCR and the 2(-Delta Delta C(T)) Method. Methods. (2001) 25:402–8. 10.1006/meth.2001.1262 11846609

[21] Polman CH Reingold SC Banwell B Clanet M Cohen JA Filippi M. Diagnostic criteria for multiple sclerosis: 2010 revisions to the McDonald criteria. Ann Neurol. (2011) 69:292–302. 10.1002/ana.22366 21387374 PMC3084507

[22] Sie C Korn T Mitsdoerffer M. Th17 cells in central nervous system autoimmunity. Exp Neurol. (2014) 262 (Pt A):18–27. 10.1016/j.expneurol.2014.03.009 24681001

[23] Luchtman DW Ellwardt E Larochelle C Zipp F. IL-17 and related cytokines involved in the pathology and immunotherapy of multiple sclerosis: current and future developments. Cytokine Growth Factor Rev. (2014) 25:403–13. 10.1016/j.cytogfr.2014.07.013 25153998

[24] Tian L Lappalainen J Autero M Hanninen S Rauvala H Gahmberg CG. Shedded neuronal ICAM-5 suppresses T-cell activation. Blood. (2008) 111:3615–25. 10.1182/blood-2007-09-111179 18223167

[25] Khalil M Teunissen CE Otto M Piehl F Sormani MP Gattringer T. Neurofilaments as biomarkers in neurological disorders. Nat Rev Neurol. (2018) 14:577–89. 10.1038/s41582-018-0058-z 30171200

[26] Kuhle J Kropshofer H Haering DA Kundu U Meinert R Barro C. Blood neurofilament light chain as a biomarker of MS disease activity and treatment response. Neurol Mar. (2019) 92:e1007–15. 10.1212/WNL.0000000000007032 30737333 PMC6442011

[27] Varhaug KN Barro C Bjornevik K Myhr KM Torkildsen O Wergeland S. Neurofilament light chain predicts disease activity in relapsing-remitting MS. Neurology Neuroimmunol Neuroinflamm. (2018) 5:e422. 10.1212/nxi.0000000000000422 29209636 PMC5707445

[28] Disanto G Barro C Benkert P Naegelin Y Schadelin S Giardiello A. Serum neurofilament light: a biomarker of neuronal damage in multiple sclerosis. Ann Neurol. (2017) 81:857–70. 10.1002/ana.24954 28512753 PMC5519945

[29] Kuhle J Barro C Disanto G Mathias A Soneson C Bonnier G. Serum neurofilament light chain in early relapsing remitting MS is increased and correlates with CSF levels and with MRI measures of disease severity. Multi Scler. (2016) 22:1550–9. 10.1177/1352458515623365 26754800

[30] Siller N Kuhle J Muthuraman M Barro C Uphaus T Groppa S. Serum neurofilament light chain is a biomarker of acute and chronic neuronal damage in early multiple sclerosis. Multi Scler. (2018) [Epubh ahead of print]. 10.1177/1352458518765666 29542376

[31] Preische O Schultz SA Apel A Kuhle J Kaeser SA Barro C. Serum neurofilament dynamics predicts neurodegeneration and clinical progression in presymptomatic Alzheimer's disease. Nat Med. (2019) 25:277–83. 10.1038/s41591-018-0304-3 30664784 PMC6367005

[32] Schmal H Czermak BJ Lentsch AB Bless NM Beck-Schimmer B Friedl HP. Soluble ICAM-1 activates lung macrophages and enhances lung injury. J Immunol. (1998) 161:3685–93. 9759893

[33] Jankowska KI Williamson EK Roy NH Blumenthal D Chandra V Baumgart T. Integrins modulate T cell receptor signaling by constraining actin flow at the immunological synapse. Front Immunol. (2018) 9:25. 10.3389/fimmu.2018.00025 29403502 PMC5778112

[34] Brosnan CF Cannella B Battistini L Raine CS. Cytokine localization in multiple sclerosis lesions: correlation with adhesion molecule expression and reactive nitrogen species. Neurology. (1995) 45(6 Suppl. 6):S16–21. 7540265 10.1212/wnl.45.6_suppl_6.s16

[35] Yoshihara Y Oka S Nemoto Y Watanabe Y Nagata S Kagamiyama H. An ICAM-related neuronal glycoprotein, telencephalin, with brain segment-specific expression. Neuron. (1994) 12:541–53. 7794412 10.1016/0896-6273(94)90211-9

[36] Ning L Tian L Smirnov S Vihinen H Llano O Vick K. Interactions between ICAM-5 and beta1 integrins regulate neuronal synapse formation. J Cell Sci. (2013) 126 (Pt 1):77–89. 10.1242/jcs.106674 23015592 PMC3603512

[37] Nutile-McMenemy N Elfenbein A Deleo JA. Minocycline decreases *in vitro* microglial motility, beta1-integrin, and Kv1.3 channel expression. J Neurochem. (2007) 103:2035–46. 10.1111/j.1471-4159.2007.04889.x 17868321

[38] Paetau S Rolova T Ning L Gahmberg CG. Neuronal ICAM-5 inhibits microglia adhesion and phagocytosis and promotes an anti-inflammatory response in LPS stimulated microglia. Front Mol Neurosci. (2017) 10:431. 10.3389/fnmol.2017.00431 29311819 PMC5743933

[39] Di Battista AP Buonora JE Rhind SG Hutchison MG Baker AJ Rizoli SB Blood biomarkers in moderate-to-severe traumatic brain injury: potential utility of a multi-marker approach in characterizing outcome. Front Neurol. (2015) 6:110 10.3389/fneur.2015.00110 26074866 PMC4443732

