# Supplementary material for: Neuronal ICAM-5 Plays a Neuroprotective Role in Progressive Neurodegeneration
Source: Front Neurol. 2019 Mar 12;10:205. doi: 10.3389/fneur.2019.00205 (PMC6422935; doi:10.3389/fneur.2019.00205)

Supplementary Figure 1: Correlation of age and ICAM-5 levels. Correlation of age and ICAM-5 levels in healthy donors (HD), RRMS, PPMS, SPMS and pooled patients revealed no significant correlations.

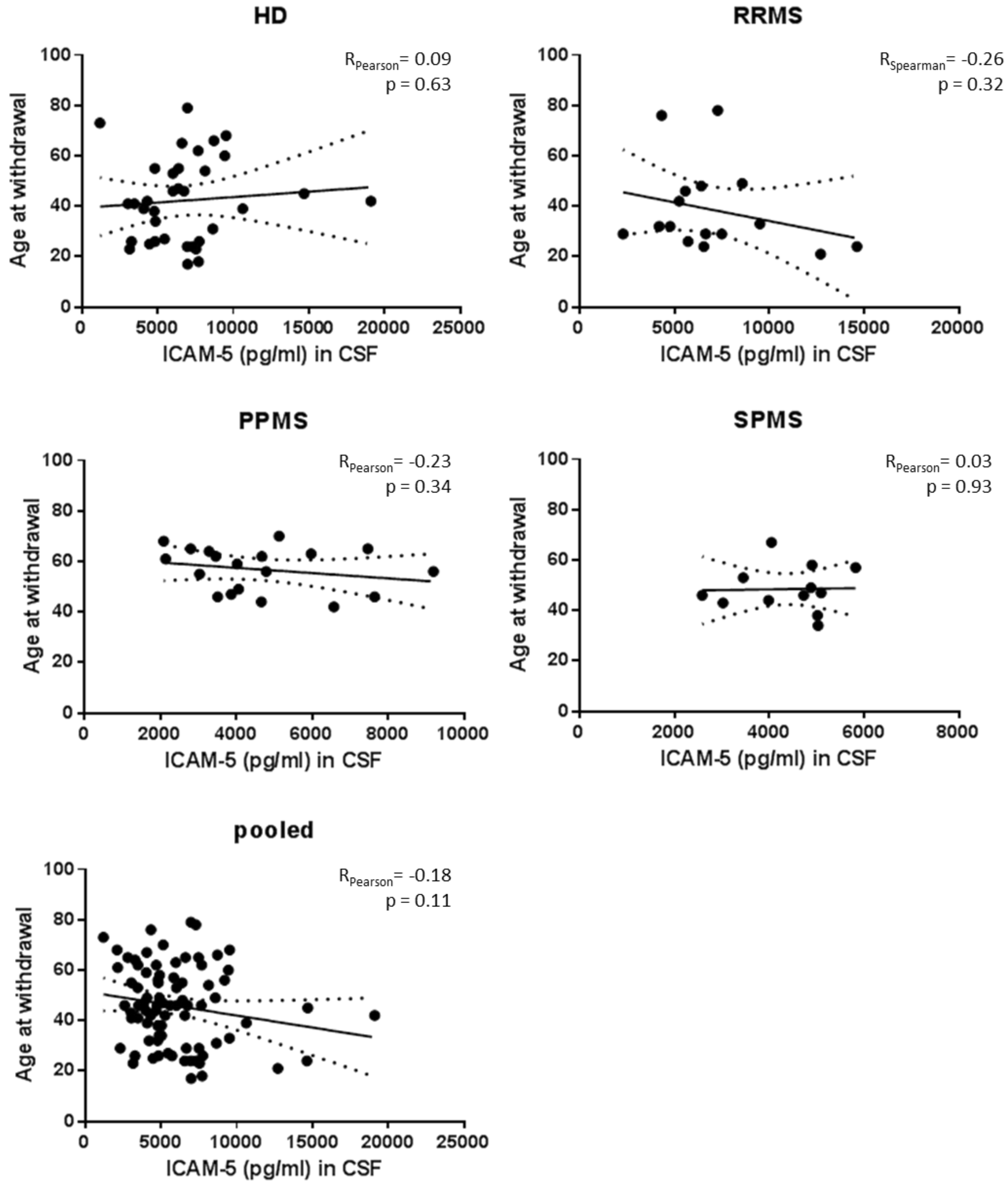

Supplement: Supplementary file 1 [file Data_Sheet_1.PDF]
